# Baicalein alleviates osteoarthritis by protecting subchondral bone, inhibiting angiogenesis and synovial proliferation

**DOI:** 10.1111/jcmm.16538

**Published:** 2021-05-03

**Authors:** Bin Li, Kaizhe Chen, Niandong Qian, Ping Huang, Fangqiong Hu, Tao Ding, Xing Xu, Qi Zhou, Bo Chen, Lianfu Deng, Tianwen Ye, Lei Guo

**Affiliations:** ^1^ Shanghai Key Laboratory for Prevention and Treatment of Bone and Joint Diseases Shanghai Institute of Traumatology and Orthopaedics Ruijin Hospital Shanghai Jiao Tong University School of Medicine Shanghai China; ^2^ Department of Orthopaedic Surgery Changzheng Hospital Second Military Medical University Shanghai China

**Keywords:** angiogenesis, baicalein, osteoarthritis, subchondral bone, synovitis

## Abstract

Osteoarthritis (OA) is one of the most frequent chronic joint diseases with the increasing life expectancy. The main characteristics of the disease are loss of articular cartilage, subchondral bone sclerosis and synovium inflammation. Physical measures, drug therapy and surgery are the mainstay of treatments for OA, whereas drug therapies are mainly limited to analgesics, glucocorticoids, hyaluronic acids and some alternative therapies because of single therapeutic target of OA joints. Baicalein, a traditional Chinese medicine extracted from *Scutellaria baicalensis* Georgi, has been widely used in anti‐inflammatory therapies. Previous studies revealed that baicalein could alleviate cartilage degeneration effectively by acting on articular chondrocytes. However, the mechanisms involved in baicalein‐mediated protection of the OA are not completely understood in consideration of integrality of arthrosis. In this study, we found that intra‐articular injection of baicalein ameliorated subchondral bone remodelling. Further studies showed that baicalein could decrease the number of differentiated osteoblasts by inhibiting pre‐osteoblasts proliferation and promoting pre‐osteoblasts apoptosis. In addition, baicalein impaired angiogenesis of endothelial cells and inhibited proliferation of synovial cells. Taken together, these results implicated that baicalein might be an effective medicine for treating OA by regulating multiple targets.

## 1 INTRODUCTION

Osteoarthritis (OA) is the most prevalent degenerative joint disease in older adults, resulting in severe pain and disability.^1^ Pathologically, OA is characterized by degeneration of articular cartilage (AC), sclerosis of subchondral bone, marginal osteophyte formation and synovial inflammation.^2^ Currently, one major hurdle to treat OA is a complete understanding of the mechanisms that drive the pathological progression of the disease.^3^ Previously, AC degeneration has long been considered as primary factor initiating OA development and numerous treating strategies responsible for AC degeneration have been applied to alleviate the progression of OA.^4^, ^5^, ^6^ However, recent researches revealed that targeting AC alone may not be sufficient to halt the disease progression and the ‘arthrosis is an organ’ concept has begun to be recognized as a fundamental basic science as our understanding of the pathophysiology of OA grows.^3^, ^7^, ^8^ Growing evidence suggested that both subchondral bone and synovium were also actively involved in OA development and often preceded AC damage.^9^, ^10^ Treatment approaches focussed on AC degeneration, subchondral bone abnormalities and synovitis together have been promised to be new choices for OA.^11^, ^12^

Subchondral bone is a bone area below the AC and formed by the subchondral plate, which is a layer of highly vascular cortical bone.^13^ Subchondral bone sclerosis and osteophyte formation were associated with the abnormal phenotype and increased anabolism of the osteoblasts.^14^, ^15^, ^16^ Additionally, studies have shown that osteogenesis accompanied with abnormal subchondral bone angiogenesis may contribute to the development of subchondral bone remodelling. Thus, osteoblasts and vascular endothelial cells can act as key targets for OA treatment.^17^

Baicalein is a flavonoid extracted from *Scutellaria baicalensis* Georgi (HuangQin in Chinese), a Chinese medical plant used in anti‐inflammatory and anti‐allergic therapies frequently.^18^, ^19^ Simultaneously, baicalein has been revealed to alleviate the OA disease effectively by ameliorating apoptotic and catabolic phenotype of chondrocytes in AC.^20^, ^21^ However, the mechanisms involved in baicalein‐mediated protection of the OA are not completely understood in consideration of integrality of arthrosis.

In this study, we found that intra‐articular injection of baicalein ameliorated subchondral bone remodelling. Further studies showed that baicalein could decrease the number of differentiated osteoblasts by inhibiting pre‐osteoblasts proliferation and promoting pre‐osteoblasts apoptosis. In addition, baicalein impaired angiogenesis of human umbilical vein endothelial cells (HUVECs) and inhibited proliferation of fibroblast‐like synovial cells (FLSs). Taken together, these results implicated that baicalein might be an effective medicine for treating OA through by regulating multiple targets.

## 2 MATERIALS AND METHODS

### 2.1 Animal experiments

Adult male Sprague–Dawley rats were used to induce OA model by destabilized medial meniscus (DMM) surgery (three groups and in each group n = 5; eight weeks old; mean body weight = 220 g). Briefly, after anaesthetisation, a medial articular incision approach was made to expose the right knee joint. Then, the medial meniscus ligament was transected, and the medial meniscus was dissociated gently. Finally, the medial capsular incision was sutured, and the skin was closed. A sham operation was performed by only opening the joint cavity. The skin wound healed 1 week after DMM surgery, and a total of 1 mg baicalein (Cayman Chemicals) per knee were intra‐articular injected once weekly for 10 consecutive weeks, whereas injected saline was used as control. The usage of baicalein is in line with the product instruction. First, baicalein was dissolved in DMSO at storage concentrations (20 mg/mL). Baicalein is sparingly soluble in aqueous; therefore, we apply Tween‐80 as co‐solubiliser. The detailed proportion is as follows: baicalein (100 μL, 20 mg/mL) + normal saline (800 μL) + Tween‐80 (100 μL). Injected dose is 50 μL solution per knee. All animal experiments were performed according to the protocol approved by the Shanghai Jiao Tong University (SJTU) Animal Care and Use Committee.

### 2.2 Radiological evaluation

Knees of OA rats were received to radiographical evaluation at 12 week after surgery. The intact knee joints were obtained and fixed in 70% ethanol for 24 hours after killing by excessive anaesthesia. Samples were scanned using SkyScan1172 high‐resolution micro‐CT (Bruker) as previously described,^20^ with some modifications that set the parameters as follows: 100 kVp, 100 μA and 10.0 μm per pixel. The data were reconstructed and three‐dimensional modelled for further analysis.

### 2.3 Histomorphometry and immunofluorescence

Samples were fixed in 4% paraformaldehyde overnight, followed by decalcification in EDTA‐buffered saline solution (pH 7.4, 0.25 mol/L) for 21 days. Tissue sections were then cut longitudinally to obtain 10 μm sections. Histological changes and subchondral bone content were observed by HE and Safranin‐O/Fast Green staining. Immunofluorescence staining was applied by standard protocol. We incubated the sections with Runx2 (1:200, ab76956, Abcam), Osterix (1:200, ab22552, Abcam) and CD31 (1:200, NB100‐2284, Novus) antibodies followed by fluorescence‐linked secondary antibodies. Fluorescence images were acquired by using the Laser scanning confocal microscopy (LSM800, ZEISS).

### 2.4 Osteoblast culture

Osteoblasts were isolated from the calvaria of neonatal Sprague–Dawley rats by collagenase digestion following the previous protocols.^21^ Briefly, dissected calvarium from neonatal rats and removed periosteum and blood vessels to reduce other cells. Excised bone chips into approximate 1‐2 mm^3^ pieces and digest fibrous tissue with 0.25% trypsin at 37°C. Digest bone chips in 10 mL PBS containing 0.1% Collagenase I and 0.05% trypsin for 1 hour in a shaking incubator with a shaking speed of 200 rpm. Collect the released cells by centrifugation for 5 minutes at 1000 rpm. Suspend the cells in 10 mL of complete alpha minimum essential medium (α‐MEM, Gibco Laboratories) containing 10% foetal bovine serum (FBS, Gibco Laboratories) and 100 µg/mL penicillin–streptomycin solution (HyClone) at 37°C in a humidified incubator containing 5% CO_2_.

### 2.5 Osteogenic differentiation

Osteoblasts (2.5 × 10^4^ cells/well) were plated in a 24‐well plate. Osteogenic differentiation of osteoblasts carried out using osteogenesis induction medium containing α‐MEM media supplemented with 50 μg/mL ascorbic acid, 10^−8^ mol/L dexamethasone, and 10 mmol/L β‐glycerophosphate (Sigma).^22^, ^23^ Every other day, we changed osteogenesis induction medium.

### 2.6 Fibroblast‐like synovial cells culture

The FLSs were isolated from synovial tissues from 12 weeks rat joints following the previous protocols.^24^, ^25^ Synovial tissues obtained from knee joints of eight weeks male Sprague–Dawley rats were rinsed with phosphate‐buffered solution (PBS) 2‐3 washes and sectioned into pieces of about 1 mm^3^ then transferred to culture flasks containing Dulbecco's modified Eagle's medium (DMEM, Gibco Laboratories, Invitrogen) supplemented with 10% foetal bovine serum (FBS, Gibco Laboratories). Next, the culture flask was placed upright with 5% CO_2_ at 37°C for tissue adhesion. After 4 hours, cell culture flasks were carefully laid flat. FLSs migrated out from tissue explants and grown into a monolayer of 95% confluency within three weeks. The culture medium was renewed every 2‐3 days. The FLSs from passage 3‐6 were used for further analysis.

### 2.7 TUNEL analysis in vitro and in vivo

TUNEL staining was performed using an apoptosis detection kit (Roche) following the manufacturer's instructions to identify apoptotic cells in vitro and in vivo. In brief, cells were first fixed with 4% paraformaldehyde for 30 minutes at room temperature and then permeabilised with 0.1% TritonX‐100. The tissue sections were dewaxed, hydrated, incubated with a methanol solution containing 0.2% H_2_O_2_ for 15 minutes to block endogenous peroxidase activity. The cells and sections were then fixed in proteinase K for 30 minutes and stained with TUNEL mixture for 60 minutes. The total number of osteoblasts was calculated using DAPI staining. Finally, the cells and sections were observed with a confocal microscopy.

### 2.8 CCK‐8 assay

The effects of baicalein on osteoblasts and synovial cells viability were determined by a CCK‐8 assay (Dojindo Laboratories). Briefly, osteoblasts and synovial cells were inoculated into 96‐well plates.100 µL of WST‐8 was added into each well of the plates at 37°C for 1 hour. The absorbance of each sample was measured on microplate reader (Infinite F50, Tecan) at a wavelength of 450 nm.

### 2.9 5‐Ethynil‐2′‐deoxyuridine (EdU) proliferation assay

Osteoblasts and synovial cells were inoculated into 24‐well plates and incubated with 50 μmol/L EdU (Sigma‐Aldrich) for 2 and 12 hours, respectively. Next, cells were fixed and permeabilised at room temperature. After washing the cells three times with PBS, 100 μL of 1X Apollo reaction mixture was added to each well for 30 minutes. At last, the cells were stained with Hoechst 33258. The EdU rates were calculated as the ratio of EdU‐positive cells (red cells) to total Hoechst 33342‐positive cells (blue cells).

### 2.10 Flow cytometric analysis

Flow cytometry was applied to measure the apoptosis rate of osteoblasts treated with baicalein in different concentrations (0, 2.5, 5, 10, 20, 50 μmol/L). Apoptotic and necrotic cells were analysed by using an Annexin V FITC/Propidium Iodine (PI) Apoptosis Detection Kit (Beyotime Biotechnology) following the manufacturer's instructions. Briefly, a total of 5 μL FITC‐Annexin V and 10 μL PI were added to the cell suspension tube and incubated for 30 minutes at room temperature in a light proof way. Cells were then analysed by flow cytometry. The apoptotic and necrotic cell rates were obtained.

### 2.11 Quantitative real‐time PCR (qRT‐PCR)

Total RNA from osteoblasts was extracted using TRIzol reagent (TaKaRa). Total RNA was synthesised into cDNA by a RevertAid First Strand cDNA Synthesis Kit (TaKaRa) and applied to qRT‐PCR using the SYBR Premix Ex Tag Kit (TaKaRa). Relative gene expression was calculated using 2^−ΔΔCt^ method. The primer sequences used in this study are described in Table 1.

**TABLE 1:** Primer sequences used for qRT‐PCR in this study

Gene	Primer	Sequence	Organism
GAPDH	FORWARD REVERSE	AAACCCATCACCATCTTCCA GTGGTTCACACCCATCACAA	Rat
Alp	FORWARD REVERSE	AGGCAGGATTGACCACGG GCTCACCATGGGAGCCAGAC	
Runx2	FORWARD REVERSE	GGCCTTCAAGGTTGTAGCCC CCCGGCCATGACGGTA	
OCN	FORWARD REVERSE	GGGCCTTTGCTTTCCATATT CAGTGGCATTAACCAACACG	

### 2.12 Western blotting analysis

Western blotting analysis was accomplished according to a previous report.^26^ Total protein from each group was fractionated by 10% SDS‐PAGE and electroblotted onto a nitrocellulose membrane followed by blocking with 5% non‐fat milk and incubated with primary antibodies against Caspase3 (1:500, ab13847, Abcam), Bcl‐2 (1:500, ab194583, Abcam), Runx2(1:500, ab76956, Abcam), OCN(1:300, sc‐390877, Santa Cruz Biotechnology, Inc) and β‐actin (1:1000, 3700, Cell Signaling Technology) followed by incubation with a HRP‐conjugated secondary antibodies (1:4000, Santa Cruz) at room temperature for 1 hour and visualized by enhanced chemiluminescence Western blot system.

### 2.13 Tube formation

Matrigel (BD Biosciences) was dissolved at 4°C and coated 100 μL Matrigel in 48‐well plates after incubating at 37°C overnight. HUVECs (3 × 10^4^) were cultured in 200 μL media. After 6 hours incubation at 37°C, cells were fixed and permeabilised at room temperature. At last, tube formation was stained using phalloidin and DAPI to visualise tube branches and length clearly under a confocal microscopy.

### 2.14 Matrigel plug assay

Matrigel plug angiogenesis was applied as previously described.^27^ HUVECs treated with 0, 10, 20 and 50 μmol/L baicalein resuspended with 300 μL Matrigel and subcutaneously injected into four‐week‐old male nude mice. After seven days, Matrigel pellets were acquired, partially fixed with 4% formalin, embedded in paraffin, then processed HE staining.

### 2.15 Alkaline Phosphate (ALP) and alizarin red staining

At the differentiation day 7, cells were washed with PBS twice, fixed with 4% formaldehyde in PBS for 30 seconds, rinsed with deionized water and stained with Alkaline Phosphatase kit (Sigma) in dark. For alizarin red staining, cells were washed with PBS twice, stained with 40 mmol/L of Alizarin red solution (pH 4.2) for 10 minutes at room temperature and washed with deionized water. The images of stained cells were captured under a microscope.

### 2.16 Statistical analysis

The composite data are expressed as mean ± SD Statistical analysis was performed with two‐sided Student's *t* test and one‐way analysis to show the difference between groups. Differences were considered be significant at *P* < .05.

## 3 RESULTS

### 3.1 Intra‐articular injection of baicalein ameliorates subchondral bone remodelling in OA

**FIGURE 1:**
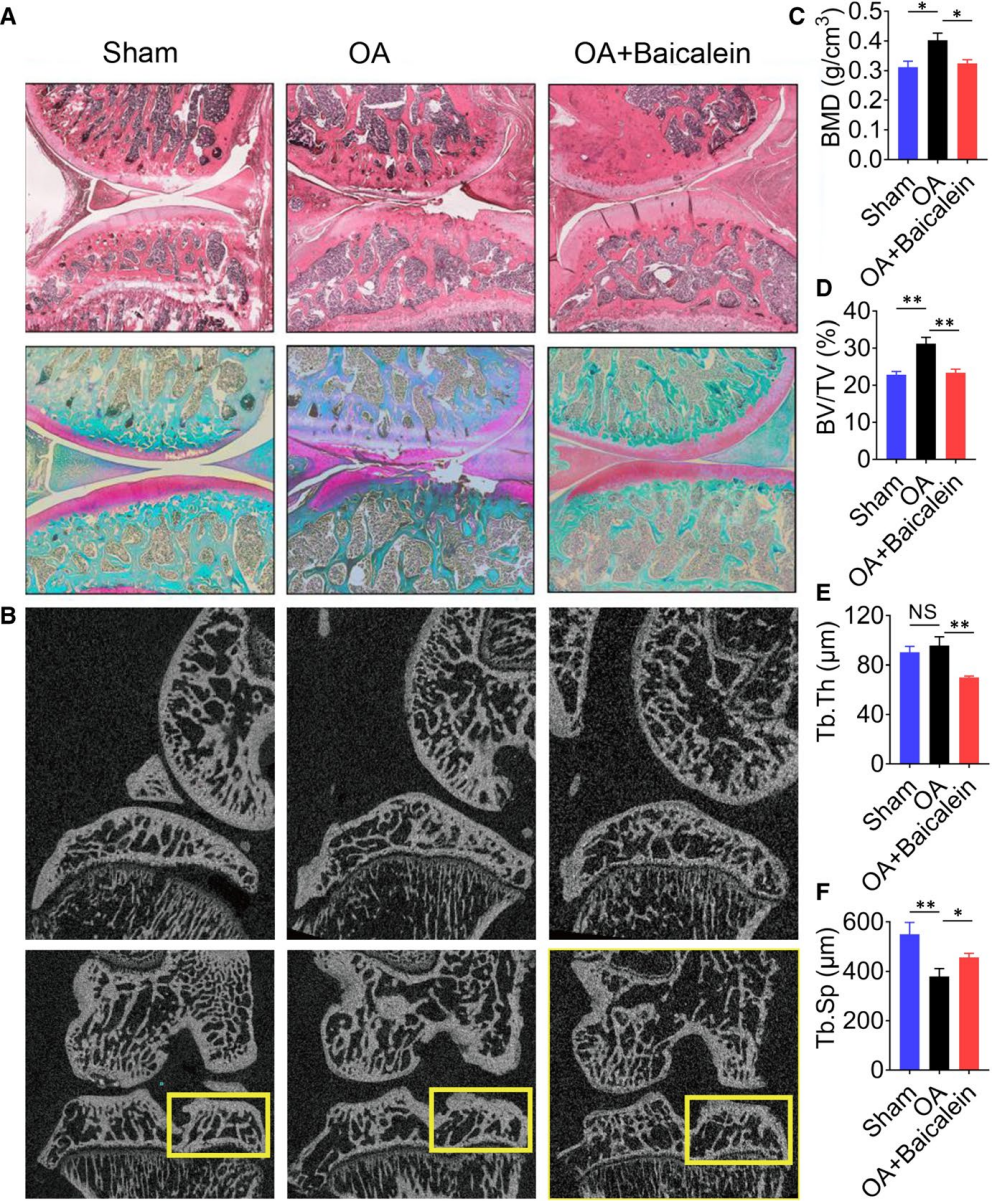
Intra‐articular injection of baicalein ameliorates subchondral bone remodelling in OA. A, Representative H&E staining and safranin O staining of the articular cartilage and subchondral bone after treating osteoarthritis employing baicalein. B, The effect of baicalein on the structure of tibial subchondral bone analysed by micro‐CT. C‐F, Baicalein reduced tibial subchondral bone mineral density (BMD), bone volume fraction (BV/TV), trabecular thickness (Tb.Th) and increased trabecular separation(Tb.Sp) compared to DMM surgery groups (**P < .01, *P < .05)

H&E and safranin O staining showed the cartilage integrity and subchondral bone histological patterns in sham, OA and baicalein‐injected OA group. Baicalein could ameliorate the progression of OA induced by DMM surgery effectively (Figure 1A). Furthermore, the effect of baicalein on the structure of tibial subchondral bone was analysed by micro‐CT (Figure 1B). As shown in Figure 1C‐F, baicalein significantly reduced the tibial subchondral bone mineral density (BMD), bone volume fraction (BV/TV), trabecular thickness (Tb.Th) and increased trabecular separation (Tb.Sp) compared to OA counterparts (all *P* < .05). The reconstructed results and subchondral bone microstructure demonstrated that the injection of baicalein could effectively ameliorate sclerosis of subchondral bone.

### 3.2 Baicalein decreases the differentiation of pre‐osteoblasts in the subchondral bone of OA

**FIGURE 2:**
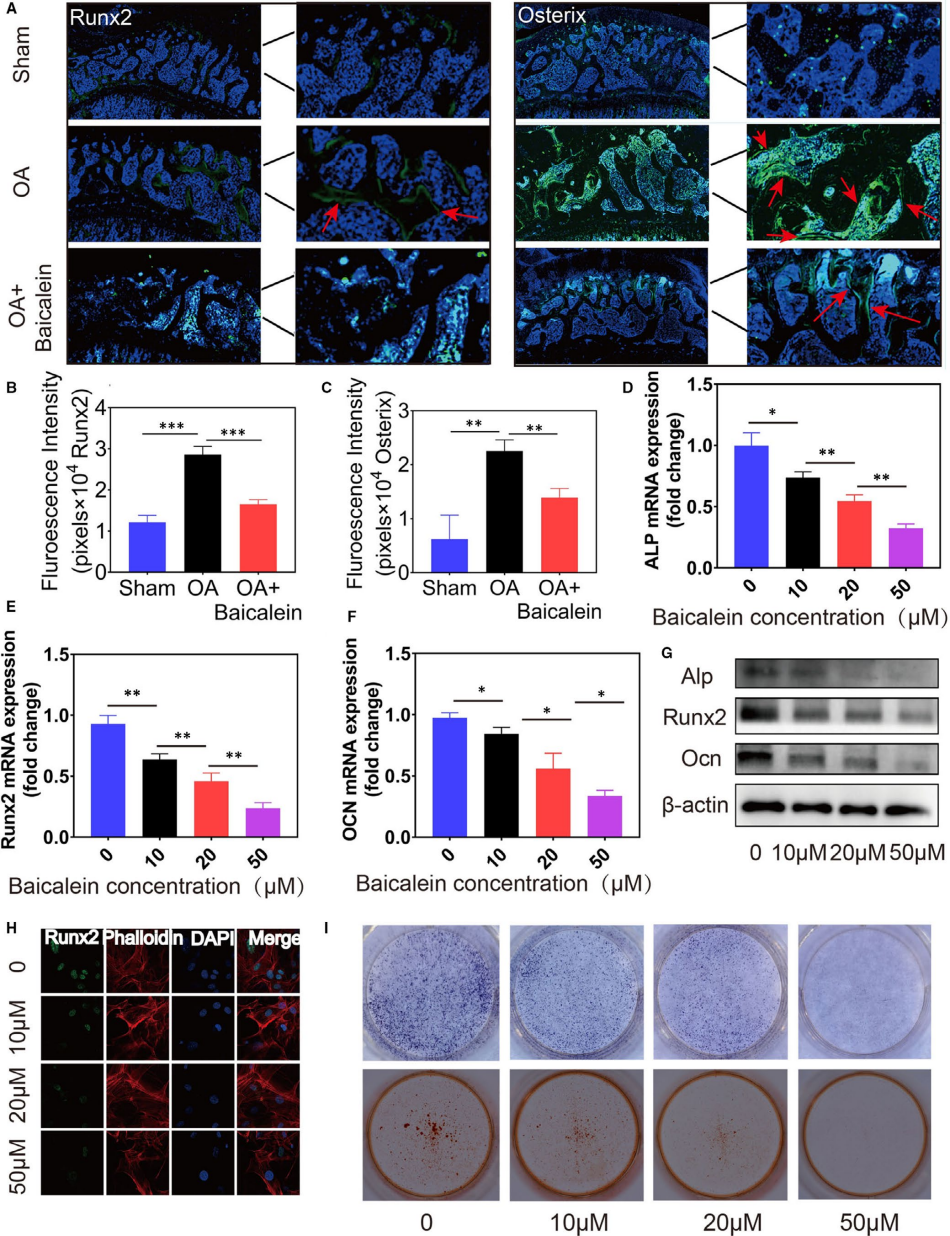
Baicalein decreases the differentiation of pre‐osteoblasts in the subchondral bone of OA. A, Immunofluorescence staining (Runx2, Osterix) visualised baicalein obviously reduced osteogenic ability of pre‐osteoblasts in subchondral bone compared to DMM surgery group. B,C, Quantification of the intensity of Runx2 and Osterix signal in subchondral bone of the tibia, n = 5, ***P < .001, **P < .01. D‐G, The qRT‐PCR analyses indicated the mRNA expression of ALP, Runx2 and OCN, n = 3, ***P < .001, **P < .01, *P < .05. G, Western blotting analysis of ALP, Runx2 and OCN expression, β‐actin was used as reference gene. H, Immunofluorescence assay results showing the expression level of Runx2. I, ALP staining and alizarin red staining indicated that baicalein decreases pre‐osteoblasts differentiation

To investigate whether baicalein is functional for the development of aberrant subchondral bone remodelling during OA progression, immunofluorescence staining of Runx2 and Osterix was applied to visualise the osteogenic ability of pre‐osteoblasts in subchondral bone. The results demonstrated that baicalein obviously reduced osteogenesis in subchondral bone compared to OA group (Figure 2A‐C). The qRT‐PCR and Western blot results (Figure 2D‐G) showed that the high concentrations of baicalein (10, 20, 50 μmol/L) could reduce the expression of osteogenic marker genes (Alp, Runx2, OCN). Moreover, the results of immunofluorescence assay also showed that the expression level of Runx2 (green) was decreased in osteoblasts treated with baicalein at different concentrations (Figure 2H). In addition, ALP staining and alizarin red staining (Figure 2I) indicated that baicalein could significantly decrease pre‐osteoblasts osteogenesis. All results above consistently revealed that baicalein at high concentrations effectively inhibited the differentiation of pre‐osteoblasts.

### 3.3 Baicalein induces apoptosis and decreases proliferation of pre‐osteoblasts in vivo and in vitro

**FIGURE 3:**
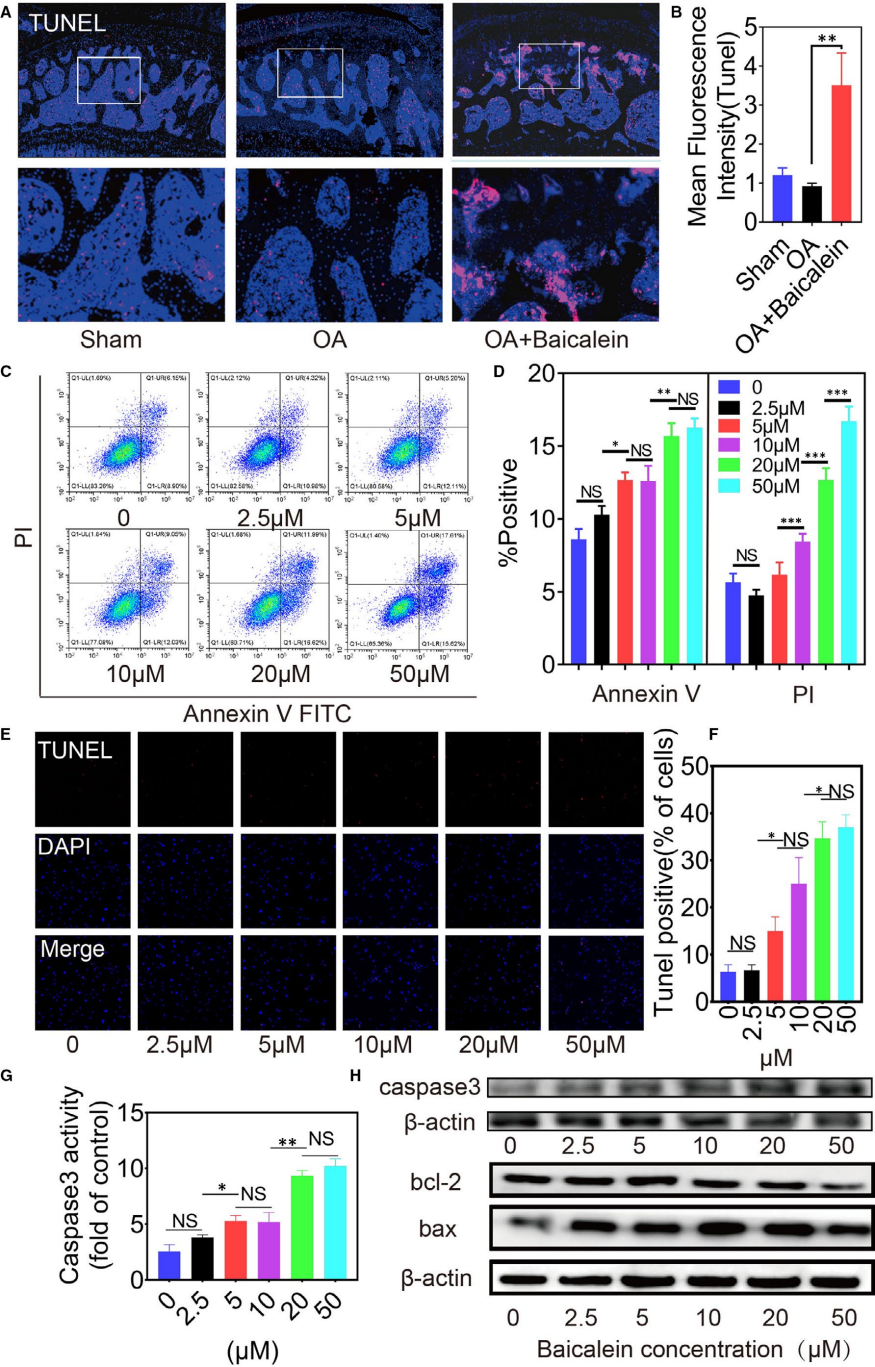
Baicalein induces apoptosis of pre‐osteoblasts in vivo and in vitro. A,B, TUNEL staining and quantification intensity of joints longitudinal sections were performed to identify apoptotic cells in vivo after injected baicalein, n = 5, **P < .01. C, Flow cytometry assay showed apoptotic cell percentage markedly increased in baicalein group than control group. D, The results of flow cytometric analysis are expressed as percentages of positive mean values ± SD, n = 3, ***P < .001, **P < .01, *P < .05. E,F, TUNEL staining and positive cell rates of pre‐osteoblasts were applied to show apoptotic cells in vitro. G, Caspase‐3 activity of pre‐osteoblasts after treated with baicalein in different concentrations, n = 3, **P < .01, *P < .05. H, Western blotting results of caspase‐3, bcl‐2 and bax

**FIGURE 4:**
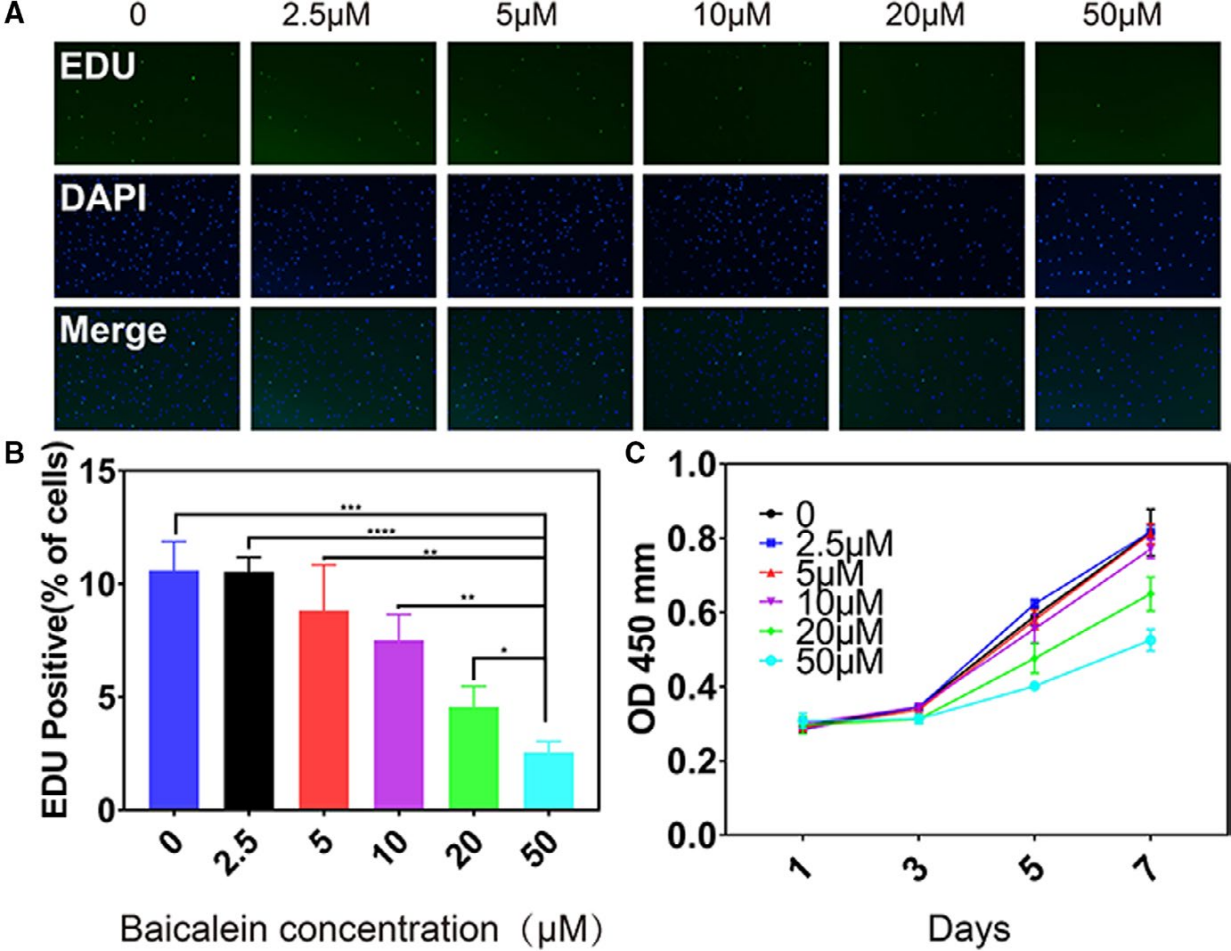
Baicalein decreases proliferation of pre‐osteoblasts in vivo and in vitro. A,B, EdU assay and corresponding statistical data at different baicalein concentrations demonstrated that baicalein decreased pre‐osteoblast proliferation, n = 3, ***P < .001, **P < .01, *P < .05. C, CCK‐8 assay revealed that baicalein induced a notable inhibition of cell viability compared with scramble control, n = 3

TUNEL staining was performed to identify apoptotic cells in vivo and in vitro. Intra‐articular injected baicalein evidently induced apoptosis in subchondral bone (Figure 3A,B), as evidenced by the increasing of TUNEL‐positive cells. Pre‐osteoblasts were treated with baicalein at concentrations of 0, 2.5, 5, 10, 20 and50 μmol/L for 24 hours and then measured for cell apoptotic rate and proliferative ability. Flow cytometry assay showed that the apoptotic cell percentage was markedly increased in the baicalein‐treated group than in the control group (Figure 3C,D). Similar results were obtained by TUNEL staining (Figure 3E,F). Additionally, as presented in Figure 3G,H, baicalein resulted in a remarkable enhancement of caspase‐3 activity. Consistently, compared with negative control, baicalein‐treated group obtained a marked increase of pro‐apoptosis protein Bax expression and a noteworthy reduction of anti‐apoptosis protein Bcl‐2 expression in pre‐osteoblasts (Figure 3H). In summary, these results suggested that baicalein induced apoptosis in pre‐osteoblasts. Additionally, EdU assay visualised by fluorescence microscope and corresponding statistical data further demonstrated that baicalein decreased pre‐osteoblast proliferation in a concentration‐dependent manner (Figure 4A,B). Moreover, CCK‐8 assay revealed that baicalein induced a notable inhibition of cell viability compared with scramble control (Figure 4C). Taken together, these results indicated that baicalein could hamper pre‐osteoblast proliferation effectively.

### 3.4 Baicalein impairs angiogenesis of subchondral bone in vivo and in vitro

**FIGURE 5:**
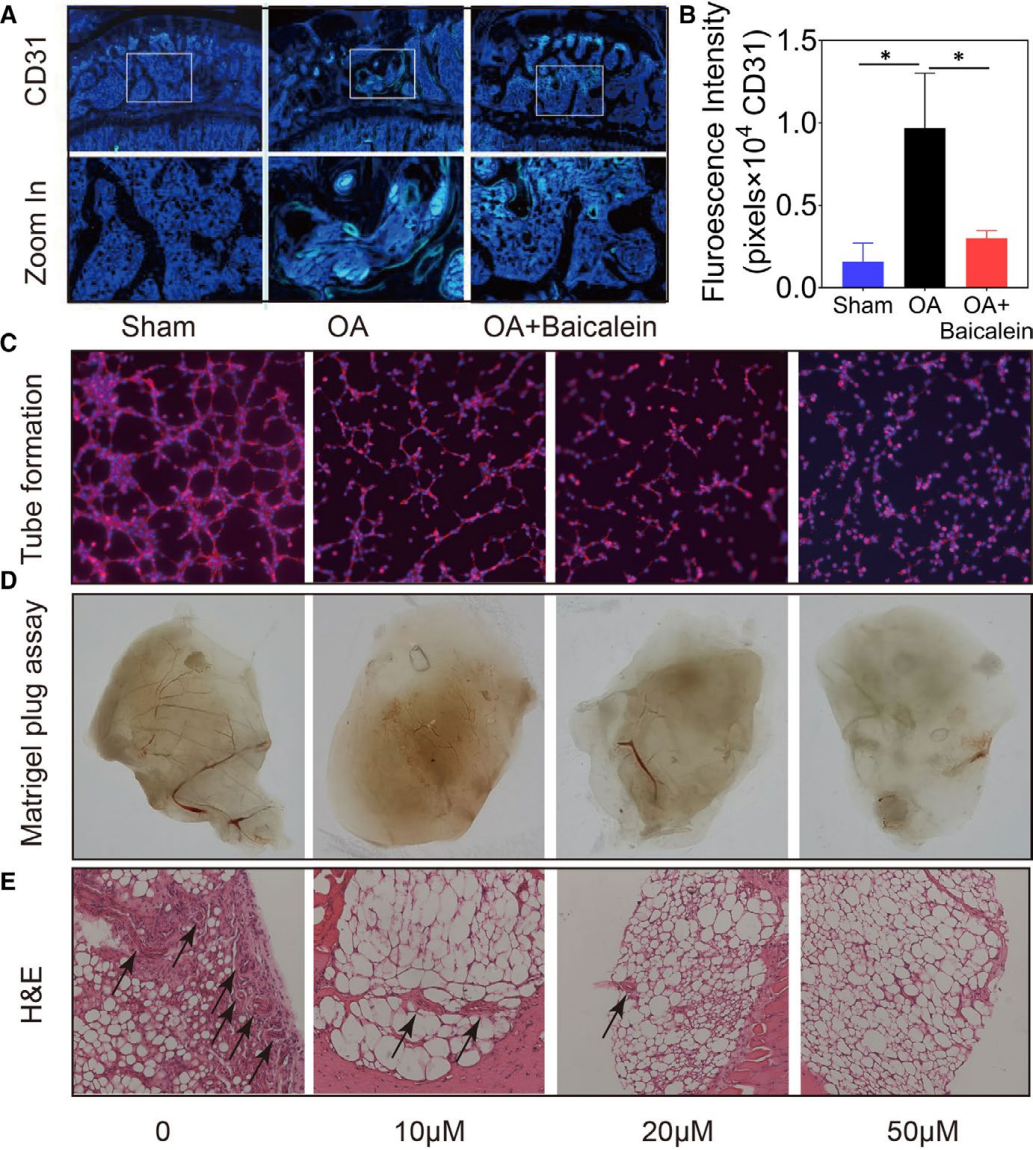
Baicalein impairs angiogenesis of subchondral bone in vivo and in vitro. A,B, Immunofluorescence staining (CD31) and quantification intensity of joints longitudinal sections visualized baicalein obviously reduced angiogenesis in subchondral bone compared to DMM surgery group, n = 5, *P < .05. C, Tube formation assay evaluated angiogenesis effects of baicalein in vitro. D,E, Matrigel plug formation and HE staining of Matrigel sections show baicalein can impair angiogenesis obviously

In OA, angiogenesis plays an important role in osteophyte development, subchondral bone remodelling and cartilage mineralization. We then detected angiogenesis in subchondral bone of OA rats. The number and percentage area of new blood vessels (CD31+) were increased in subchondral bone/bone marrow in OA group, whereas the neo‐vessel formation in baicalein‐injected group was decreased in osteoarthritic joints (Figure 5A,B). Effects of baicalein on angiogenesis in vitro were evaluated by tube formation assay. HUVECs were treated with baicalein at different concentrations and cultured in 48‐well plates with 100 μL matrigel in each well. The results showed that baicalein obviously reduced tube formation in number and length in a concentration‐dependent manner (Figure 5C). We also analysed matrigel plug formation following subcutaneous implantation in vivo. After implanted seven days, matrigel pellets were obtained. Matrigel mixed with baicalein‐treated HUVECs decreased blood vessel growth (Figure 5D) and HE staining of matrigel sections showed the similar results (Figure 5E). In summary, baicalein can obviously impair angiogenesis of subchondral bone in OA.

### 3.5 Baicalein inhibits proliferation of FLSs

**FIGURE 6:**
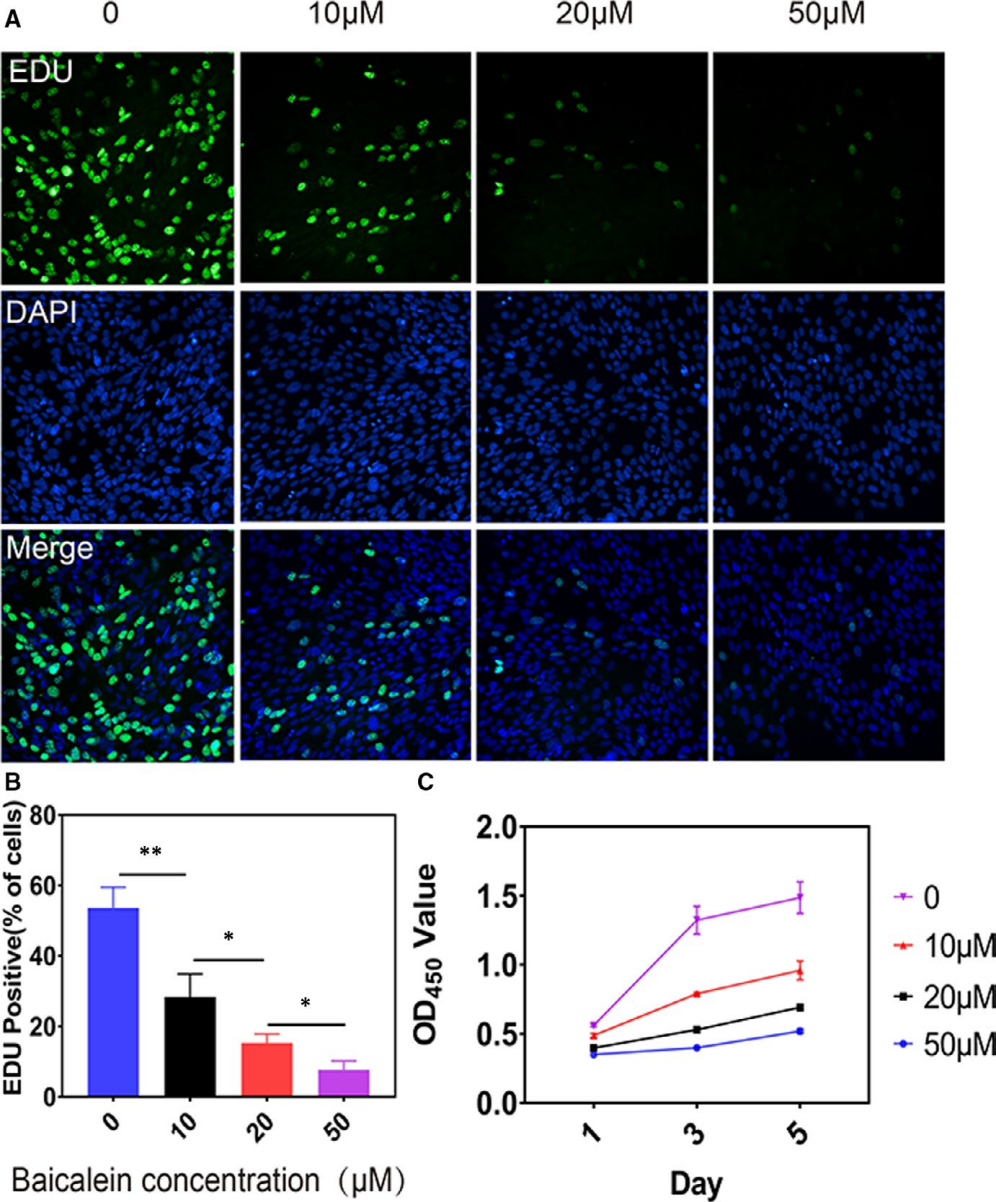
Baicalein inhibits proliferation of synovial cells. A,B, EdU assay and corresponding statistical data demonstrated that baicalein decreased proliferation n = 3, **P < .01, *P < .05. C, CCK‐8 assay revealed that baicalein induced an inhibition of FLSs compared with control, n = 3

There is increasing recognition that hyperplasia of the synovium plays an important role in the pathogenesis of OA. Thus, we next observed the effect of baicalein on FLSs. FLSs were treated with 0, 2.5, 5, 10, 20 and 50 μmol/L baicalein for 24 hours, followed by the measurement of cell proliferative rate. EdU assay showed that baicalein decreased FLSs proliferation in a concentration‐dependent manner by fluorescence microscope (Figure 6A,B). In addition, CCK‐8 assay revealed that baicalein induced inhibition of cell proliferation compared with control group (Figure 6C). Taken together, these results indicated that baicalein could inhibit synovial cells proliferation.

## 4 DISCUSSION

Although some progress has been made in the pathogenesis and therapeutic options of OA, OA is still a major obstacle of human health with high morbidity. Previous studies showed that baicalein exerted anti‐osteoarthritic properties via reducing MMPs activities and expressions in chondrocytes.^28^ Nevertheless, the effects of baicalein on other components in joint capsule remain to be unelucidated. In this study, we provided new evidence that baicalein might alleviate progression of OA by regulating subchondral bone sclerosis, aberrant angiogenesis and synovitis.

Accumulating evidence revealed that subchondral bone acts as a key factor in the pathogenesis of OA and might be an important target for the treatment of OA.^29^ During the progression of OA, subchondral bone is the site of numerous dynamic morphological transformations because of an altered osteoblast metabolism, which is part of the pathological process. In the present study, we found that intra‐articular injection of baicalein ameliorated subchondral bone remodelling by decreasing differentiation of osteoblasts (Runx2, Osterix) in vivo. Furthermore, baicalein effectively suppressed the differentiation of osteoblasts at higher concentrations (10‐50 μmol/L). These results were inconsistent with previous studies that baicalein could induce osteoblasts differentiation at low concentrations (0‐10 μmol/L). There are many reasons to explain this phenomenon. First, the concentration of baicalein used in our study was higher than that of previous studies, suggesting that baicalein with different concentrations may have different roles in the regulation of osteoblasts differentiation. Second, our results showed that baicalein decreased the number of pre‐osteoblasts by inducing pre‐osteoblasts apoptosis and inhibiting pre‐osteoblasts proliferation, which result in the decrease of the amounts of mature osteoblasts which have already differentiated. Our study demonstrated for the first time that baicalein alleviated OA through inhibiting bone formation of subchondral bone.

Abnormal vascular formation in subchondral bone is also one of the characteristic pathological features of OA.^30^ OA is thought to aggravate by osteochondral angiogenesis where blood vessels grow into the tidemark at the osteochondral junction.^31^ Consistently, studies have shown that aberrant subchondral bone angiogenesis accompanied with osteogenesis may contribute to the development of subchondral bone sclerosis and osteophyte formation.^32^ Hence, subchondral bone angiogenesis emerged in sight as a fresh therapeutic target to delay the progress of OA. Baicalein has been revealed reducing angiogenesis in different circumstances, such as inflammatory microenvironment and tumour tissues.^33^, ^34^ In this study, the effect of baicalein on vascularisation of HUVECs was tested in vivo and in vitro and the results showed that baicalein could effectively suppress angiogenesis. Thus, our study provides a new mechanism that baicalein ameliorates subchondral bone remodelling through inhibiting abnormal vascular formation and generates an outstanding approach in managing subchondral bone aberrant angiogenesis.

Vascular growth is also increased in the synovium of OA joints and associated with synovitis.^35^ Therefore, the inhibition of vascularization by baicalein is beneficial to synovium inflammation simultaneously. Furthermore, FLSs are the major effector cells that lead to synovitis.^36^ The imbalance of FLSs proliferation and apoptosis has long been considered as initiating factor for rheumatoid arthritis (RA).^37^, ^38^ Recently, the characteristic of FLSs in OA attracts more attention and numerous researches elucidate the mechanism of synovitis during OA progression.^39^ Baicalein can powerfully suppress the proliferation of FLSs to alleviate the synovitis during OA progression. In general, baicalein may mitigate synovitis by means of inhibition of vascular growth and FLSs proliferation both.

Baicalein has historically been used in anti‐oxidant, anti‐virus, anti‐bacteria, anti‐inflammatory and anti‐allergic therapies.^40^ Previously, baicalein has been revealed to alleviate OA effectively by ameliorating apoptotic and catabolic phenotypes of chondrocytes.^41^ Nevertheless, we elucidated the effect of baicalein on pre‐osteoblasts, HUVECs and FLSs. In conclusion, our study confirmed for the first time that baicalein alleviated OA by ameliorating subchondral bone remodelling. Further studies found that baicalein could regulate subchondral bone remodelling by inhibiting pre‐osteoblasts differentiation and proliferation, and inducing pre‐osteoblasts apoptosis. In addition, baicalein could suppress vascularisation to alleviate subchondral bone sclerosis and restrain synovial cells proliferation to control synovitis. Taken together, these results indicated that baicalein might be a hypothetical candidate for the treatment of OA and inspire a new approach to develop potential therapeutic agents treating degenerative joints as a whole organ.

## CONFLICT OF INTEREST

The authors declare that they have no competing interests.

## AUTHOR CONTRIBUTIONS

**Bin Li:** Conceptualization (equal); Methodology (equal); Software (lead); Writing‐original draft (lead); Writing‐review & editing (lead). **Kaizhe Chen:** Data curation (lead); Software (equal); Writing‐review & editing (equal). **Niandong Qian:** Data curation (equal); Software (equal). **Ping Huang:** Methodology (equal); Software (equal); Validation (equal). **Fangqiong Hu:** Visualization (lead). **Tao Ding:** Conceptualization (equal); Methodology (equal). **Xing Xu:** Methodology (equal); Software (equal). **Qi Zhou:** Validation (equal); Visualization (equal). **Bo Chen:** Methodology (equal); Software (equal). **Lianfu Deng:** Methodology (equal); Validation (equal). **Tianwen Ye:** Conceptualization (lead); Funding acquisition (equal); Methodology (lead); Supervision (lead). **Lei** **Guo:** Funding acquisition (lead); Project administration (lead); Supervision (lead); Writing‐review & editing (equal).

## Data Availability

All data included in this study are available upon request by contact with the corresponding author
